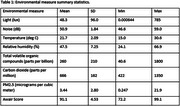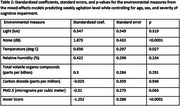# Indoor Physical Environmental Quality Is Associated with Agitation in Older Adults Living with Cognitive Impairment

**DOI:** 10.1002/alz70863_110590

**Published:** 2025-12-23

**Authors:** Wan‐Tai Michael Au‐Yeung, Lyndsey M. Miller, Allison Lindauer, Zachary T Beattie, Nora Mattek, Joel S. Steele, Jeffrey A Kaye

**Affiliations:** ^1^ Oregon Health & Science University, Portland, OR USA; ^2^ NIA‐Layton Aging & Alzheimer's Disease Center, Portland, OR USA; ^3^ Oregon Center for Aging & Technology (ORCATECH), Portland, OR USA; ^4^ NIA‐Layton Aging & Alzheimer's Disease Research Center, Portland, OR USA; ^5^ University of North Dakota, Grand Forks, ND USA

## Abstract

**Background:**

Agitation is a challenging behavior exhibited by older adults with cognitive impairment, causing distress in caregivers, earlier placement into long‐term care, and faster disease progression. Non‐pharmacological treatments, such as environmental modifications, should be used as first‐line treatments. The MODERATE (Monitoring Dementia‐Related Agitation Using Technology Evaluation) study aims to identify precipitants (behavioral or environmental) of agitation.

**Method:**

Older adults living in the greater Portland, Oregon, USA area were recruited into the study. Inclusion criteria were a diagnosis of cognitive impairment, currently residing with a family caregiver within their own home, and caregiver endorsement of symptom(s) of agitation, irritability/lability, motor disturbance, nighttime behaviors and/or disinhibition per Neuropsychiatric Inventory‐Questionnaire. Weekly agitation levels of the participants were reported by family caregivers through modified Cohen‐Mansfield Agitation Inventory‐Short Form questionnaires as part of weekly online surveys. Lighting, noise level, temperature, relative humidity, and air quality (CO2, PM2.5 and VOC) in their bedrooms were measured by a commercial multi‐domain environmental sensor device (Awair Omni). The Awair Omni also provides an overall environmental quality score (Awair Score) based on temperature, relative humidity, and air quality with a higher Awair Score indicating better quality. Eight mixed‐effects models were built with caregiver‐reported weekly agitation levels as the outcome, each with one of weekly mean Awair Omni measures as a predictor while controlling for age, sex, and severity of cognitive impairment.

**Result:**

Data from 10 dyads were analyzed. Mean age (SD) for people living with cognitive impairment (PLwCI) and their caregivers were 75.9 (9.2) years old and 72.3 (7.8) years old respectively. All dyads were heterosexual, and 80% of PLwCI were males. The mean (SD) monitoring period was 52.6 (29.5) weeks. In the order of decreasing effect sizes, noise, the Awair Score, and temperature were significantly associated with caregiver‐reported weekly agitation levels. See Table 1 for the summary statistics of the environmental measures and Table 2 for mixed‐effects model results.

**Conclusion:**

Multiple indoor environmental quality parameters were associated with weekly agitation in PLwCI. In order to help mitigate agitation in PLwCI, it may be important to investigate improving air quality and other environmental features holistically through intervention studies.